# Biochemical Scoring System for diagnosing Nonalcoholic Steatohepatitis

**DOI:** 10.5005/jp-journals-10018-1201

**Published:** 2016-12-01

**Authors:** Mohammad Noor-E-Alam, Shahinul Alam, AKM Khorshed Alam, Mamun Al Mahtab, Salimur Rahman

**Affiliations:** 1 Department of Hepatology, Shaheed Suhrawardy Medical College Hospital, Dhaka, Bangladesh; 2 Department of Hepatology Bangabandhu Sheikh Mujib Medical University, Dhaka, Bangladesh

**Keywords:** Biochemical scoring, Hepatic fibrosis, Nonalcoholic steatohepatitis, Noninvasive.

## Abstract

Noor-E-Alam M, Alam S, Alam AKMK, Al Mahtab M, Rahman S. Biochemical Scoring System for diagnosing Nonalcoholic Steatohepatitis. Euroasian J Hepato-Gastroenterol 2016;6(2):202-204.

Dear Editor,

The incidence and prevalence of nonalcoholic fatty liver disease (NAFLD) has reached to epidemic proportions around the world. Nonalcoholic fatty liver disease encompasses a spectrum of conditions ranging from simple steatosis to steatohepatitis, advanced fibrosis, and end-stage liver diseases. Despite the high prevalence of reported NAFLD, it still remains underdiagnosed, especially in majority of developed and resource-constrained countries.^1-3^ The gold standard of diagnosis of NAFLD is dependent on the availability of a liver biopsy specimen, but, i.e., usually nonfeasible leading to checking for noninvasive diagnostic approach of NAFLD.^4-6^ The extent of pathological process of NAFLD is shown by NAFLD activity score (NAS). On the contrary, NAFLD is also associated with alterations of several blood parameters. Patients with NAFLD exhibit increased triglyceride (T), elevated alanine aminotransferase (A), AST/ALT ratio (A), and gamma-gamma-glutamyl transferase (G). We undertook a preliminary study to assess if a scoring system of T, A, A, and G (TAAG) may have some diagnostic importance to assess NAS in NAFLD patients.

It was an observational, cross-sectional study and carried out at the Department of Hepatology, Bangabandhu Sheikh Mujib Medical University (BSMMU), Dhaka, Bangladesh over a period of 2 years. A total of 43 patients with NAFLD were enrolled in this analysis. Patient’s inclusion criterion was ultrasonographic evidence of fatty liver and exclusion criteria were significant alcohol intake (more than 20 g/day), viral hepatitis [hepatitis B virus (HBV), hepatitis C virus (HCV)], pregnancy, comorbid conditions [chronic obstructive pulmonary disease (COPD), chronic respiratory failure (CRF), cardiac failure], hypothyroidism, consumption of drugs causing fatty change in liver (steroid, oral contraceptive pill, tamoxifen, amiodarone, diltiazem, protease inhibitor). All data were collected from structured questionnaire and analyzed by Statistical Package for the Social Sciences (SPSS) 16 software. Qualitative data was analyzed by chi-square test and quantitative data by Student’s t-test. The p value below 0.05 is considered as statistically significant. The NAS score was evaluated based on the published article. The TAAG was calculated after assessing T, A, A, and G in their sera and providing each of the parameters a score (fasting serum triglyceride > ULN, ALT > ULN, AST/ALT ratio (AAR) ≤ 1 and GGT > ULN). An association of TAAG score of 3 was evaluated with NAS score of each patient.

The study enrolled 43 patients (26 females, 17 males). The body mass index (BMI) and waist circumference was calculated according to Western Pacific Region Office of World Health Organization (WHO) 2000 criteria and International Diabetes Federation 2006 criteria for the South Asians respectively. We grouped the study population (n = 43) into non-NASH fatty liver (NNFL) and nonalcoholic steatohepatitis (NASH). Non-NASH fatty liver was present in 23 patients and NASH was present in 20 patients (Tables 1 and 2).

**Table 1:** **Table 1:** Baseline characteristics of study populations (n = 43)

*Variables*		*Mean ± SD*	
Age (years)		41.1 ± 10.5	
Weight (kg)		67.8 ± 10.3	
Height (cm)		157.5 ± 8.6	
Body mass index (kg/m^2^)		27.3 ± 3.0	
Waist circumference (cm)		93.0 ± 6.9	
Systolic blood pressure (mm Hg)		120.0 ± 21.7	
Diastolic blood pressure (mm Hg)		80.7 ± 8.2	
Alanine aminotransferase (U/L)		48.6 ± 25.4	
Aspartate aminotransferase (U/L)		40.0 ± 25.0	
AST/ALT (AAR)		0.8 ± 0.3	
Gamma glutamyl transaminase		50.0 ± 34.9	
Fasting blood sugar (mmol/L)		5.5 ± 1.3	
2-hour ABF (mmol/L)		7.9 ± 1.7	
Total cholesterol (mg/dL)		212.5 ± 43.6	
Triglyceride (mg/dL)		253.1 ± 202.7	
High density lipoprotein (mg/dL)		37.1 ± 9.8	
Low density lipoprotein(mg/dL)		132.4 ± 39.7	

Data expressed as mean ± SD. SD: Standard deviation

**Table 2:** **Table 2:** Clinical and laboratory characteristics of study population (n = 43)

*Variables*		*NNFL (NAS 0–4) (mean ± SD)*		*NASH (NAS ≥ 5–8) (mean ± SD)*		*p-value by independent samples t-test*	
Age (years)		39.2 ± 9.5		43.2 ± 11.3		0.22	
Weight (kg)		66.7 ± 9.7		69.1 ± 11.0		0.45	
Height (cm)		156.3 ± 8.6		159 ± 8.5		0.31	
BMI (kg/m^2^)		27.4 ± 3.3		27.2 ± 2.7		0.88	
Waist circumference (cm)		92.3 ± 5.6		93.8 ± 8.3		0.53	
SBP (mm Hg)		117.1 ± 9.8		128.2 ± 15.2		0.009	
DBP (mm Hg)		79.3 ± 8.56		82.2 ± 7.69		0.24	
ALT (U/L)		46.4 ± 23.7		51.1 ± 27.7		0.55	
AST (U/L)		43.3 ± 32.1		36.1 ± 12.7		0.32	
AST/ALT		0.9 ± 0.3		0.80 ± 0.3		0.27	
GGT		46.5 ± 29.4		58.3 ± 40.2		0.28	
FBS (mmol/L)		5.2 ± 1.3		5.9 ± 1.1		0.74	
2-hour ABF (mmol/L)		7.7 ± 1.4		8.2 ± 2.0		0.43	
Total cholesterol (mg/dL)		209.8 ± 41.4		215.5 ± 46.9		0.67	
TG (mg/dL)		262.7 ± 264.6		242.1 ± 97.5		0.73	
HDL (mg/dL)		38.0 ± 8.6		36.1 ± 11.2		0.54	
LDL (mg/dL)		131.4 ± 37.5		133.4 ± 42.8		0.87	

p-value was determined by independent samples t-test

Serum triglyceride (T) level was elevated about ULN in 58.1%; serum ALT (A) level above the ULN in 25.5% NAFLD patients. The AST/ALT (A) ratio was ≤ 1 in 79.0% NAFLD patients. Serum GGT level above the ULN was 25.5% in study population. The TAAG score ≥ 3 had a sensitivity of 40% and specificity of 26.1% not significantly correlates (p = 0.33, chi-square test) to NASH prediction. The receiver operation characteristic (ROC) curve showing TAAG scoring system ≥ 3 had sensitivity 40% and specificity 26.1% to identify NAFLD.

Although this study has shown that the biochemical markers bear limited utility as a noninvasive marker of NAS scoring, ample opportunities remain to alter the entity and definition of TAAG. For example, a level of ALT above 65 IU/L was taken as ULN, but ALT levels of 42 or 30 IU/L may also be considered as ULN. Also, it remains to assess if TAAG score of 2 may have any kind of implication in NAS score or not. However, the utility of TAAG may be checked in bigger sample size.

The lack of correlation of TG levels and inflammatory markers with NAS scale in NAFLD indicates how complex the nature of fibrogenesis is in NAFLD patients. In case of hepatitis due to viral etiology, fibrosis is presumed to be due to inflammation of the liver. However, that may not be vase in case of NAFLD. Although our study failed to show a relation of TAAG with NAS score, it possibly exposes the unique nature of hepatic fibrosis during NAFLD.
